# Is it my fault? The role of the feeling of guilt in adolescent peer victimization

**DOI:** 10.3389/fpsyg.2022.1089689

**Published:** 2023-01-27

**Authors:** Celeste León-Moreno, Cristian Suárez-Relinque, Juan Evaristo Callejas-Jerónimo, Fernanda Inéz García-Vázquez

**Affiliations:** ^1^Department of Psychology and Sociology, University of Zaragoza, Teruel, Spain; ^2^Department of Education and Social Psychology, Pablo de Olavide University, Seville, Spain; ^3^Department of Statistics and Educational Research, University of Seville, Seville, Spain; ^4^Department of Education, Technological Institute of Sonora, Obregon, Mexico

**Keywords:** feeling of guilt, peer victimization, loneliness, adolescents, *ex post facto* study

## Abstract

**Introduction:**

The aim of this study was to analyze the relationships between feelings of guilt, peer victimization in school, and loneliness based on adolescents’ gender.

**Methods:**

A total of 671 Spanish students (50.7% boys), aged 10–16 years old (*M* = 13.04, *SD* = 1.80) from six public primary and secondary schools participated in the study. A Multivariate Analysis of Variance (3 × 2) was calculated.

**Results:**

Adolescents with high levels of guilt presented greater physical, verbal, and relational victimization, as well as higher levels of loneliness. In addition, boys high in guilt had the highest scores in overt physical victimization, while girls high in guilt had the highest levels of loneliness.

**Discussion:**

Results obtained suggest that adolescents with greater feelings of guilt feel responsible for being victims of peer aggression and for feeling lonely. These findings suggest the need to address the feeling of guilt, taking into account the gender perception.

## Introduction

Peer victimization in schools is defined as a type of abuse where students are subjected to physical, verbal, and psychological violence by one or more peers (Graham, 2006). Previous studies have found a positive association between victimization and psychosocial adjustment problems, such as high levels of loneliness (León-Moreno et al., 2019; Cava et al., 2021), higher social anxiety (Webb et al., 2021; Wu et al., 2021), lower popularity, more social integration problems in the classroom (Garandeau et al., 2019; Gao et al., 2021), and suicidal ideation (Lucas-Molina et al., 2018; Quintana-Orts et al., 2020). These particularly painful and stressful interpersonal experiences for the adolescent can lead students to ask: Why me? Is it my fault? Thus, this study explores the links between feelings of guilt, peer school victimization, and loneliness in adolescence.

### Feelings of guilt and school victimization

Guilt is defined as an unpleasant feeling towards oneself due to the perception of responsibility and regret about a harm caused (real or imagined; Tilghman-Osborne et al., 2012; Conejero et al., 2019). According to Misailidi and Kapsali (2020), the feeling of guilt can emerge from the awareness that the adolescent has broken a social or moral norm (e.g., “Believing that one has done something wrong”), and, also, due to the awareness of what others think or believe about the wrong behavior (e.g., “Others believe that I intended to do something wrong”). In this regard, the feeling of guilt encourages the repair of damaged relationships (Vaish et al., 2016), and strengthens interpersonal bonds by inhibiting actions that jeopardize group relationships (Gazzillo Fimiani et al., 2020). Therefore, according to some authors, the moderate presence of this feeling is positive and adaptive by fostering the development of moral and prosocial behaviors (Hoffman, 1982; Roos et al., 2014). However, other authors (Giammarco and Vernon, 2015; Zahn-Waxler and Schoen, 2016) postulated that the feeling of guilt becomes inappropriate and excessive when it is based on cognitive distortions or erroneous beliefs regarding responsibility for a given event, such as the internal attribution of victim blaming (Thornberg et al., 2015; Harsey et al., 2017).

According to Graham and Juvonen (1998), following an episode of victimization, two internal attributions of blame may emerge: characterological and behavioral. Characterological guilt refers to the perception that negative experiences are attributed to internal, stable, and uncontrollable causes, which may inhibit the victim from seeking external help and support (Forsberg and Horton, 2020; Tholander et al., 2020), whereas behavioral guilt relates to specific controllable actions. For instance, an adolescent may attribute being victimized by a peer to being an unpleasant person (characterological guilt) or to not being kind enough that day (behavioral guilt). Prior studies have highlighted that, compared to characterological self-blame, behavioral self-blame is less maladaptive because students perceive that “things will not always be this way and can change” (Graham and Juvonen, 1998; Schacter et al., 2015).

### Feelings of guilt and loneliness

Another concerning aspect of the feeling of guilt is that it can have negative consequences in the victim’s interpersonal relationships (Valdés-Cuervo et al., 2021), leading to an aggravation of victimization situations (Schacter and Juvonen, 2015). According to the Social Information Processing Model proposed by Crick and Dodge (1994), when students face a negative interpersonal experience, they try to understand why it happened, and their subjective interpretations in turn explain their emotional reactions. Thus, as suggested by Zimmer-Gembeck et al. (2016), the feeling of guilt would have a negative impact on the expectations of support and acceptance from others, accounting for not only less involvement in their social relationships (Schacter and Juvonen, 2017; Russell et al., 2019), but also for their greater perception of loneliness (Bruno et al., 2009). Similarly, positive peer relationships, and friendships in particular, have been found to have a buffering effect on victims with respect to the negative effects of guilt (Tholander et al., 2020). Moreover, Chen and Graham (2012) stated that the mechanism underlying the buffering effect of affiliative relationships is that the positive appraisal of the supportive social network may help to displace the victim’s internal attribution of guilt. For instance, a victimized adolescent who has the support of a best friend is likely to conclude, “I get along with the good guys. This is not my fault.”

Regarding gender differences, data obtained in different studies show that, in general, the perception of guilt is higher in girls than in boys (Bennett et al., 2005; Mazzone et al., 2016). Specifically, it has been found that one of the areas in which girls report more feelings of guilt is in the interpersonal domain (Graber et al., 2016). According to Etxebarria and Perez (2003), girls infer a greater sense of guilt in interpersonal interactions because of the expectations of care and maintenance of the affective bonds in which they have been socialized. Given this background, the main objective of the present study was to analyze the relationship between feelings of guilt, peer school victimization, and loneliness in adolescents as a function of gender. Therefore, the following hypotheses were proposed:

*H1*: Adolescents with high feelings of guilt will present greater peer school victimization-physical, overt and relational-, as well as greater feelings of loneliness.

*H2*: Girls with high feelings of guilt will report greater peer school victimization-physical, overt, and relational-and greater feelings of loneliness.

## Materials and methods

### Participants

A multistage cluster sampling was carried out to select a random sample (*N* = 594) from a total population of 58,679 adolescents of both sexes between 10 and 16 years old living in the province of Cordoba. The sample consisted of 671 adolescents of both sexes (50.7% boys and 49.3% girls), aged between 10 and 16 years old (*M* = 13.04, *SD* = 1.80), enrolled in primary education (5th and 6th grades), and compulsory secondary education (ESO) in six schools, four public and two state-subsidized, in the province of Cordoba (Spanish).

### Measures

*Guilt Scale: Inappropriate and Excessive*, from Tilghman-Osborne et al. (2012). It consists of 48 items with a response range from 0 (not at all) to 3 (very much) that measures the degree of guilt experienced by the adolescent in the past year (e.g., “Imagine your class is participating in a game and your team loses. You cannot help but think they lost because of you”). The Cronbach’s Alpha obtained in the present sample was acceptable (α = 0.94).

*Peer Victimization Scale* (Mynard and Joseph, 2000), adapted to Spanish by Martínez-Ferrer et al. (2018). It consists of 25 items with a response range from 1 (never) to 4 (always) that rates how frequently the adolescent has been subjected to violent behaviors in the last year. The scale consists of three dimensions: overt physical victimization (e.g., “A peer has beaten me up”); overt verbal victimization (e.g., “A peer has insulted me”); and relational victimization (e.g., “A peer has told my secrets to others”). The Cronbach’s Alpha obtained in the present sample was acceptable (α = 0.93).

*Loneliness Scale* by Russell et al. (1980), adapted to Spanish by Expósito and Moya (1999). It is composed of 20 items with a response range from 1 (never) to 4 (always) that evaluates the degree of loneliness experienced by the adolescent in the last year (e.g., “How often do you feel isolated from others?”). The Cronbach’s Alpha obtained in the present sample was acceptable (α = 0.89).

### Procedure

First, an informative seminar was held with teachers and families to explain the objectives, the scope of the study, and the procedure to be followed. Next, the necessary authorizations were obtained from school administrators and participating families were requested to give active informed consent for their child to participate in the study. The battery of instruments was administered voluntarily, anonymously, and supervised in two different sessions of approximately 45 min during school hours. Participants were guaranteed the confidentiality of the information obtained. The study complied with the ethical values required in research with human beings, respecting the fundamental principles included in the Declaration of World Medical Association (2013).

### Data analysis

First, a two-stage cluster analysis was performed for guilt, obtaining three groups (Fernandez-Rio et al., 2014; Chen et al., 2020): low guilt (*n* = 454), medium guilt (*n* = 176), and high guilt (*n* = 41). Next, a multivariate factorial design (MANOVA, 3 × 2) was conducted with the SPSS statistical program (version 20) considering guilt (low, medium, and high) and gender (boy versus girl) as fixed factors to analyze possible interaction effects. The three dimensions of school victimization-physical, verbal, and relational-and feelings of loneliness were considered as dependent variables. Univariate tests (ANOVAS) were calculated to study differences in statistically significant variables and the Bonferroni *post-hoc* test (α = 0.05) was performed.

## Results

We examined whether the groups were similar in terms of sociodemographic variables. As shown in Table 1, according to gender, non-significant differences were found [χ^2^(2) = 0.428 *p* > 0.05].

**Table 1:** Sociodemographic variables.

		Feeling of guilt			
Variables	Total sample	Low *N* = 174	Medium *N* = 320	High *N* = 177	χ^2^
Gender					χ^2^(2) = 0.428 (n.s.)
Boys	340 (50.7%)	90 (51.7%)	164 (51.3%)	86 (48.6%)	
Girls	331 (49.3%)	84 (48.3%)	156 (48.8%)	91 (51.4%)	

χ^2^: Chi-square; n.s: non significant

### Multivariate factor analysis

In the MANOVA, statistically significant differences were found in the main effects of feelings of guilt [Λ = 0.939, *F*(8, 1,324) = 5.261, *p* < 0.001, η^2^_p_ = 0.031], and gender [Λ = 0.893, *F*(4, 662) = 19.827 *p* < 0.001, η^2^_p_ = 0.107]. In addition, a statistically significant interaction effect was obtained between feelings of guilt and gender [Λ = 0.926, *F*(8, 1,324) = 6.497, *p* < 0.001, η^2^_p_ = 0.038].

### Feelings of guilt

The ANOVA results showed significant differences in overt physical victimization, *F*(2, 668) = 9.892, *p* < 0.001, η^2^_p_ = 0.029, verbal victimization, *F*(2, 668) = 12. 709, *p* < 0.001, η^2^_p_ = 0.037, relational victimization, *F*(2, 668) = 14.762, *p* < 0.001, η^2^_p_ = 0.042, and loneliness, *F*(2, 668) = 7.854, *p* < 0.001, η^2^_p_ = 0.023. Bonferroni tests (α = 0.05) indicated that adolescents with high and medium feelings of guilt reported higher levels than adolescents with low feelings of guilt in physical, verbal, and relational victimization. Regarding loneliness, adolescents with high levels of guilt had statistically higher scores in feeling lonely than adolescents with medium and low levels of guilt.

### Demographic variable: Gender

Results of the ANOVA revealed significant differences for gender in the variables physical victimization *F*(1, 669) = 29.214, *p* < 0.001, η^2^_p_ = 0.042, and verbal victimization *F*(1, 669) = 6.387, *p* < 0.01, η^2^_p_ = 0.006. As shown in Table 2, Bonferroni tests (α = 0.05) indicated that boys, relative to girls, obtained higher scores in physical and verbal victimization (Table 3).

**Table 2:** Means, standard deviations, and differences on guilt, peer victimization, and gender.

	Feeling of guilt						Gender			
**Variables**	**Low**	**Medium**	**High**	**F(2, 668)**	**η^2^_p_**	***Post hoc***	**Boys**	**Girls**	**F(1, 669)**	**η^2^_p_**
PV	1.23^c^	1.32^b^	1.44^a^	9.892^***^	0.029	a, b > c	1.34	1.20	29.214^***^	0.042
	(0.30)	(0.39)	(0.42)				(0.37)	(0.29)		
VV	1.61^c^	1.78^b^	1.90^a^	12.709^***^	0.037	a, b > c	1.72	1.62	6.387^**^	0.006
	(0.46)	(0.55)	(0.57)				(0.48)	(0.51)		
RV	1.49^c^	1.76^b^	1.79^a^	14.762^***^	0.042	a, b > c	1.53	1.59	2.582	. 004
	(0.43)	(0.53)	(0.56)				(0.45)	(0.50)		
L	1.76^c^	1.82^b^	2.05^a^	7.854^***^	0.023	a > b, c	1.79	1.79	0.002	0.000
	(0.43)	(0.43)	(0.62)				(0.42)	(0.47)		

RV, relational victimization; PV, physical victimization; VV, verbal victimization; L, loneliness.

*** *p* < 0.001.

**Table 3:** Means, standard deviations, *F* values, and Bonferroni *post hoc* test for the guilt groups.

	Gender	Feeling of guilt			F(5, 665)	η^2^_p_	*Post hoc*
		Low	Medium	High			
PV	Boys	1.27^a^	1.43^b^	1.67^c^	13.807^***^	0.094	b > e, a, d c > f, e, a, d
		(0.30)	(0.43)	(0.49)			
	Girls	1.19^d^	1.19^e^	1.26^f^			
		(0.28)	(0.29)	(0.23)			
L	Boys	1.77^a^	1.78^a^	1.80^a^	5.578^***^	0.040	b > a
		(0.41)	(0.44)	(0.48)			
	Girls	1.76^a^	1.78^a^	2.24^b^			
		(0.44)	(0.43)	(0.65)			

PV, physical victimization; L, loneliness.

*** *p* < 0.001.

### Interaction analysis

**Figure 1:**
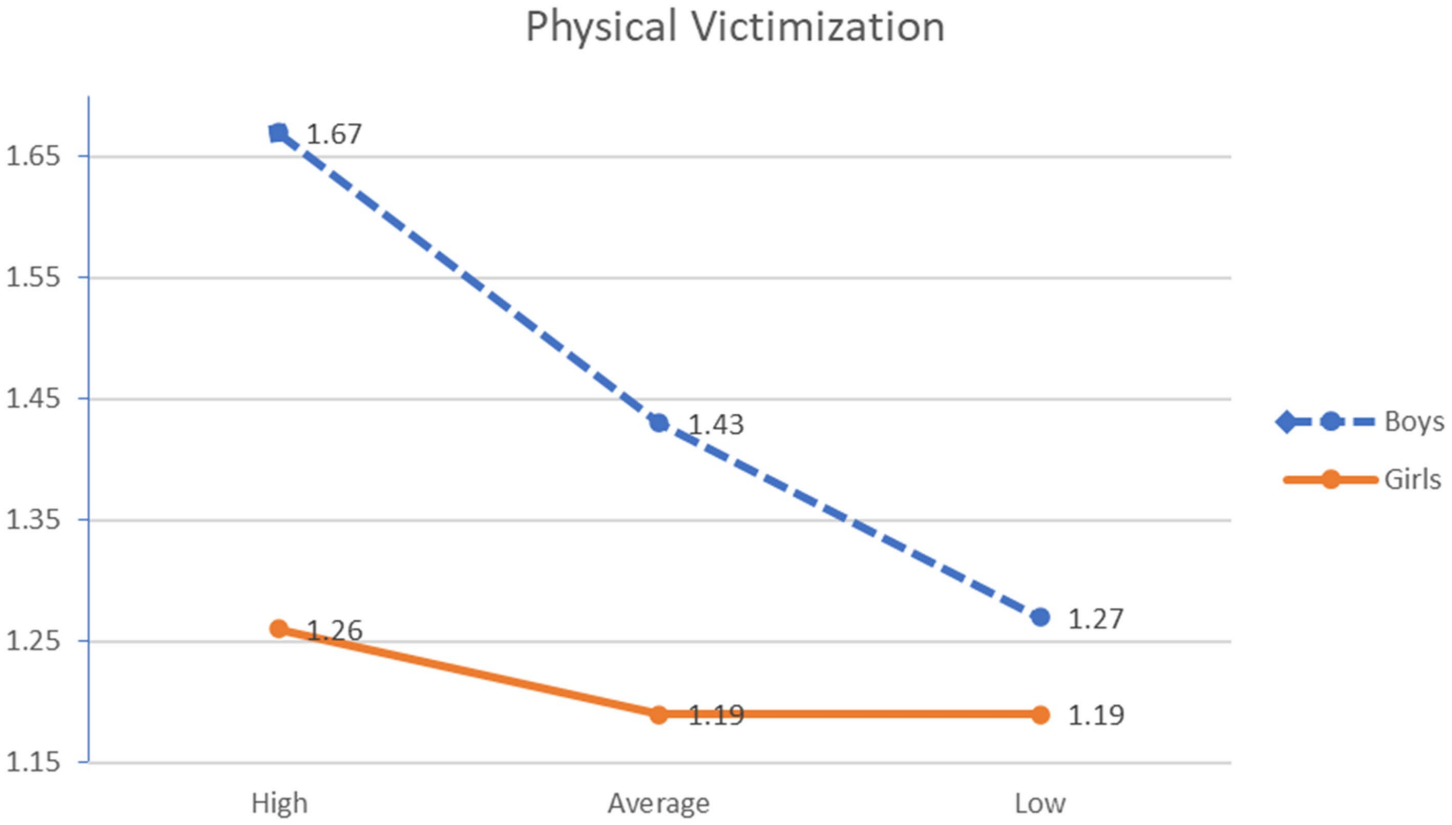
Interaction effect feeling of guilt × gender and physical victimization.

**Figure 2:**
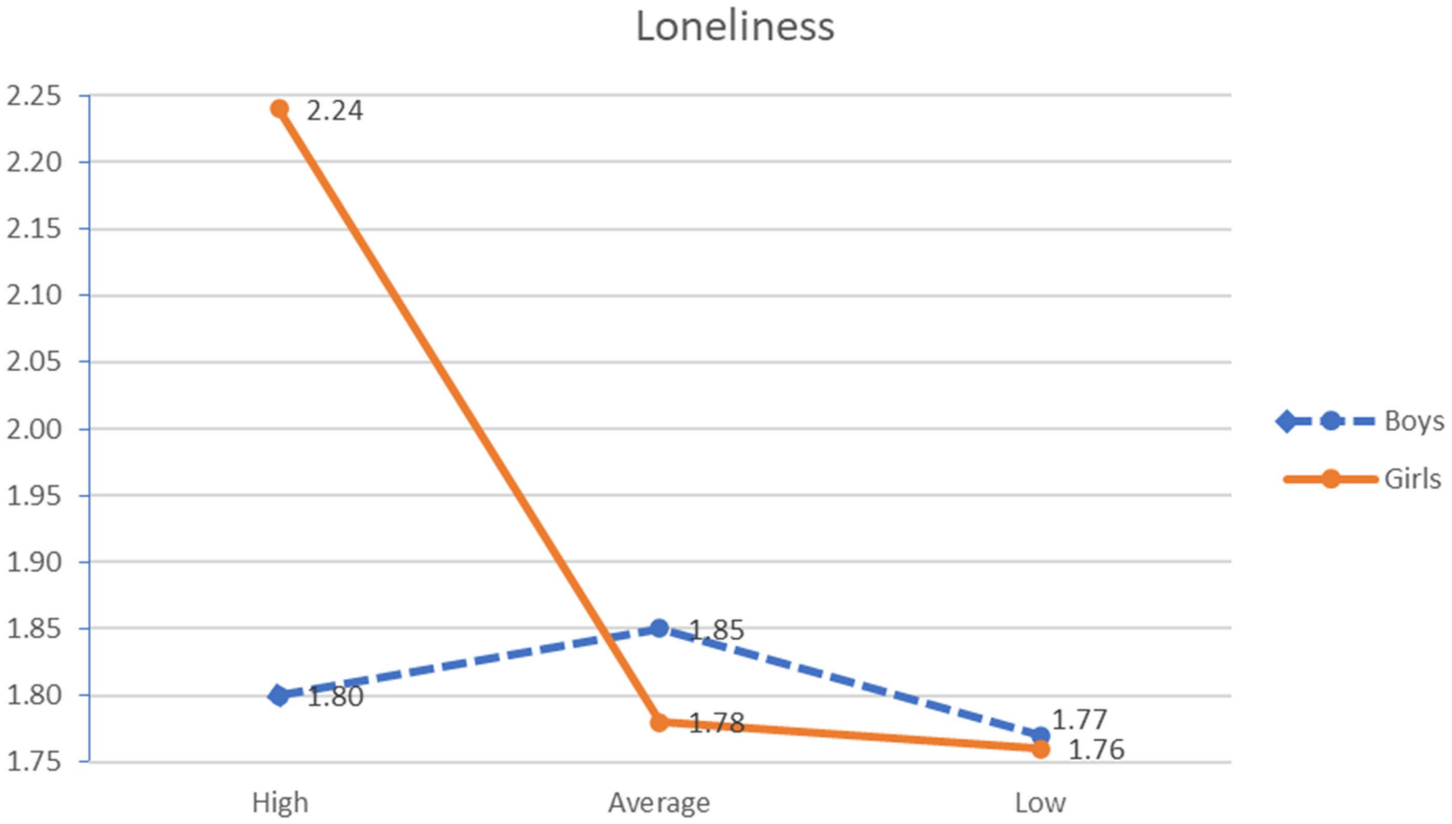
Interaction effect feeling of guilt × gender and loneliness.

Two statistically significant interaction effects were found between guilt and gender in the variables physical victimization *F*(5, 665) = 13.807, *p* < 0.001, η^2^_p_ = 0.094, and the feeling of loneliness *F*(5,665) = 5.578, *p* < 0.001, η^2^_p_ = 0.040. Regarding the first interaction, it was observed that boys with medium levels of guilt showed greater physical victimization than boys and girls with low levels of guilt and girls with medium levels of guilt. Moreover, boys with high levels of guilt reported a higher level of victimization than girls with high and medium levels of guilt and girls and boys with low levels of guilt. With respect to the second interaction, ex-post analyses revealed that girls with high levels of guilt had a greater feeling of loneliness than the rest of the groups analyzed (see Figures 1, 2).

## Discussion

The aim of the present study was to analyze the relationship between feelings of guilt, peer victimization in school, and loneliness in school-aged adolescents. First, as predicted in the first hypothesis, it was observed that adolescents with high levels of guilt presented greater school victimization-physical, overt, and relational-, which was in line with previous studies (Chen and Chen, 2019; Tholander et al., 2020). These findings are, in our view, highly relevant as various studies have pointed out that adolescents showing irrational guilt tend toward internal attribution of their victimization (Wei-Ru and Li-Ming, 2019; Forsberg and Horton, 2020), which may inhibit the search for external support (Harsey et al., 2017), and are therefore more likely to face prolonged victimization (Schacter and Juvonen, 2017). We consider these results interesting because they contribute to deepening our understanding of victim coping strategies.

In terms of loneliness, our findings are consistent to previous studies in which a positive association between guilt and loneliness was found (Bruno et al., 2009; Rostami and Jowkar, 2016). Considering that the attribution of guilt is associated with the belief or feeling of having transgressed social ethical norms or for not meeting group expectations (Etxebarria and Perez, 2003), it is plausible to think that the attribution of guilt may eventually generate an inhibition of interpersonal interactions, and undermine the adolescent’s feeling of belonging and social integration (Wei-Ru and Li-Ming, 2019; Forsberg and Horton, 2020).

Concerning the interaction effect between guilt and gender, significant differences were found in the physical victimization and loneliness variables, whereas no significant differences were found in verbal or relational victimization. Specifically, the results of the present study indicated that boys with high levels of guilt scored highest in physical victimization. This finding can be explained on the basis of Control-Mastery Theory (CMT; Weiss et al., 1986; Gazzillo et al., 2017), which postulates that guilt has an interpersonal and adaptive origin and is based on the adolescents´ need to feel that their environment values and accepts them. Thus, CMT considers guilt as a consequence of the fear of losing important relationships due to internal causes. It has been observed that physical violence is considered as an essential component of normative models of masculinity and power (Carrera-Fernández et al., 2018; Rosen and Nofziger, 2019). Therefore, it is likely that boys may attribute their victimization situation to internal causes, such as increased physical weakness, contributing to their tendency to feel guilty.

As for gender differences, it has also been observed that girls with high levels of guilt demonstrated a greater degree of loneliness than the rest of the groups analyzed. In this regard, Chen and Chen (2019) highlighted that the feeling of loneliness in adolescence may have a more negative effect on girls due to the high importance they attach to their interpersonal relationships and the belief that they have been isolated or rejected because of their own actions, increasing their tendency to blame themselves. However, contrary to expectations, there are no differences in the relationships between the feeling of guilt and verbal and relational victimization. A possible explanation for this result could be due to the normalization of these forms of violence. In a previous qualitative study (Bouchard et al., 2021), it was found that girls tend to normalize insults and behaviors aimed at damaging their reputation or social status because these actions are socially reinforced behaviors in different areas of their socialization, such as their favorite series or films. In addition, previous studies have pointed out that these behaviors are more difficult to detect and, in many cases, minimized even by the educational community itself (Bauman and Del Rio, 2006; Wójcik and Rzeńca, 2021), aspects that can hinder the victim’s self-perception (Chen and Chen, 2019), making it difficult for them to seek help (Bastiaensens et al., 2015).

Based on our findings, we suggest that these variables should be taken into account in the field of psychoeducational intervention and therapeutic work with victims at an emotional level. We recommend promoting emotional education programs because it is important for victims to be able to identify and reduce the feeling of irrational guilt and its consequences, thus facilitating proactive coping strategies, such as help-seeking and cognitive restructuring. Likewise, it is recommended that attention be paid to gender differences found in order to design prevention and intervention programs in a more specific manner.

It is important to underline that the results obtained in this study should be interpreted with caution because of the cross-sectional and correlational nature of the data. Future research incorporating the temporal dimension would help to clarify the differences obtained between the groups. Moreover, because self-reported measures were used, the measurement of the feeling of guilt, peer school victimization, and the feeling of loneliness variables, may entail some biases and social desirability effects. This limitation could be resolved by incorporating different sources of information (peer group, educational community, and family) since adolescence is a developmental period characterized by a certain degree of vulnerability and the difficulties experienced by adolescents (Bakadorova et al., 2020; Fuentes et al., 2022), not only as potential victims or aggressors in bullying and cyberbullying (Lo Cricchio et al., 2021), but also more difficulties in comparison to childhood and adulthood such as lower self-concept (Garcia et al., 2018), more problems in school (Bakadorova et al., 2020), and drug use (Fuentes et al., 2022). During adolescence, family can have a positive but also detrimental impact. Thus, when parents are involved (high warmth), children have more support and communication with them (Villarejo et al., 2020; Gimenez-Serrano et al., 2022) and benefit by achieving better adjustment (Queiroz et al., 2020; Climent-Galarza et al., 2022). School is also an important context for adolescents (Musitu-Ferrer et al., 2019; Salmela-Aro and Upadyaya, 2020). Academic motivation may be reduced, as well as performance (Veiga et al., 2021), although this trend is more marked in boys than in girls (Musitu-Ferrer et al., 2019). Overall, despite some age-related differences in academic performance (Fenzel, 1992; Salmela-Aro and Upadyaya, 2020), it has been shown that good academic performance in middle childhood and adolescence is beneficial for good adjustment (Kupersmidt and Coie, 1990; Prince and Nurius, 2014). It would also be worthwhile for future research to incorporate victims’ interpretation of shame as this variable is closely related to the feeling of guilt.

## Data availability statement

The original contributions presented in the study are included in the article/supplementary material, further inquiries can be directed to the corresponding author.

## Author contributions

All authors listed have made a substantial, direct, and intellectual contribution to the work and approved it for publication.

## Funding

This study was funded by the project: “Cyber-violence and relationships between peers: power, reputation and popularity in adolescents,” subsidised by the European Regional Development Fund (ERDF) and by the Regional Ministry of Economic Transformation, Industry, Knowledge and Universities of the Regional Government of Andalusia (“Junta de Andalucía“in Spanish), (ref P18-RT-1487).

## Conflict of interest

The authors declare that the research was conducted in the absence of any commercial or financial relationships that could be construed as a potential conflict of interest.

## Publisher’s note

All claims expressed in this article are solely those of the authors and do not necessarily represent those of their affiliated organizations, or those of the publisher, the editors and the reviewers. Any product that may be evaluated in this article, or claim that may be made by its manufacturer, is not guaranteed or endorsed by the publisher.
